# 3-(1-Methyl-3-imidazolio)propane­sulfonate: a precursor to a Brønsted acid ionic liquid

**DOI:** 10.1107/S1600536810004344

**Published:** 2010-02-13

**Authors:** W. Matthew Reichert, Paul C. Trulove, Hugh C. De Long

**Affiliations:** a Department of Chemistry, US Naval Academy, 572 M. Holloway Road, Annapolis, Maryland 21402, USA; b Air Force Office of Scientific Research, 4015 Wilson Boulevard, Arlington, Virginia 22203, USA

## Abstract

The title compound, C_7_H_12_N_2_O_3_S, is a zwitterion precursor to a Brønsted acid ionic liquid with potential as an acid catalyst. The C—N—C—C torsion angle of 100.05 (8)° allows the positively charged imidazolium head group and the negatively charged sulfonate group to inter­act with neighboring zwitterions, forming a C—H⋯O hydrogen-bonding network; the shortest among these inter­actions is 2.9512 (9) Å. The C—H⋯O inter­actions can be described by graph-set notation as two *R* ^2^_2_(16) and one *R* ^2^_2_(5) hydrogen-bonded rings.

## Related literature

For the use of functionalized ionic liquids (ILs) as Brønsted acid catalysts for organic reactions, see: Cole *et al.* (2002 ▶); Yoshizawa *et al.* (2001 ▶). The local structure of ILs is often conserved on transition from the solid state to the liquid state, see: Henderson *et al.* (2007 ▶); Reichert *et al.* (2007 ▶); Triolo *et al.* (2006 ▶). For a related structure, see: Pringle *et al.* (2003 ▶). For polymorphs of ionic liquids, see: Holbrey *et al.* (2003 ▶) and for the applications of ionic liquids, see: Plechkova & Seddon (2008 ▶).
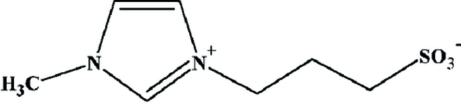


## Experimental

### 

#### Crystal data

C_7_H_12_N_2_O_3_S
*M* *_r_* = 204.25
Monoclinic, 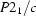

*a* = 9.8164 (4) Å
*b* = 11.7421 (5) Å
*c* = 7.9769 (3) Å
β = 94.878 (2)°
*V* = 916.13 (6) Å^3^
*Z* = 4
Mo *K*α radiation
μ = 0.33 mm^−1^
*T* = 296 K
0.29 × 0.28 × 0.13 mm

#### Data collection

Bruker SMART CCD area-detector diffractometer
Absorption correction: for a sphere (*SADABS*; Bruker, 2007 ▶) *T*_min_ = 0.910, *T*_max_ = 0.958
21327 measured reflections
8299 independent reflections
5726 reflections with *I* > 2σ(*I*)
*R*_int_ = 0.033

#### Refinement

*R*[*F* ^2^ > 2σ(*F* ^2^)] = 0.041
*wR*(*F* ^2^) = 0.120
*S* = 1.03
8299 reflections
166 parameters
All H-atom parameters refined
Δρ_max_ = 0.55 e Å^−3^
Δρ_min_ = −0.47 e Å^−3^

### 

Data collection: *SMART* (Bruker, 2007 ▶); cell refinement: *SAINT* (Bruker, 2007 ▶); data reduction: *SAINT*; program(s) used to solve structure: *SHELXS97* (Sheldrick, 2008 ▶); program(s) used to refine structure: *SHELXL97* (Sheldrick, 2008 ▶); molecular graphics: *SHELXTL* (Sheldrick, 2008 ▶); software used to prepare material for publication: *SHELXTL*.

## Supplementary Material

Crystal structure: contains datablocks I, New_Global_Publ_Block. DOI: 10.1107/S1600536810004344/kp2241sup1.cif

Structure factors: contains datablocks I. DOI: 10.1107/S1600536810004344/kp2241Isup2.hkl

Additional supplementary materials:  crystallographic information; 3D view; checkCIF report

## Figures and Tables

**Table 1:** Hydrogen-bond geometry (Å, °)

*D*—H⋯*A*	*D*—H	H⋯*A*	*D*⋯*A*	*D*—H⋯*A*
C2—H2*A*⋯O2^i^	0.923 (14)	2.197 (14)	2.9512 (9)	138.4 (12)
C5—H5*A*⋯O1^ii^	0.899 (14)	2.381 (15)	3.1573 (10)	144.6 (12)
C4—H4*A*⋯O1^iii^	0.961 (14)	2.528 (14)	3.3268 (11)	140.5 (11)
C4—H4*A*⋯O3^iii^	0.961 (14)	2.541 (14)	3.4364 (11)	155.0 (11)
C7—H7*B*⋯O3^iv^	0.961 (12)	2.613 (12)	3.1693 (10)	117.2 (9)
C8—H8*B*⋯O3^iv^	0.990 (14)	2.621 (13)	3.2086 (9)	118.1 (9)

Symmetry codes: (i) 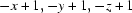; (ii) 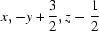; (iii) 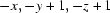; (iv) 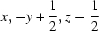.

**Fig. 1.**
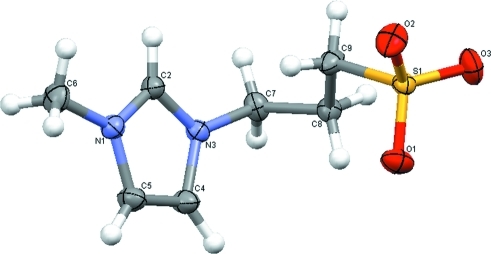
The thermal ellipsiod plot of the asymmetric unit of (I). The displacement ellipsiods are shown at the 50% probability level.

**Fig. 2.**
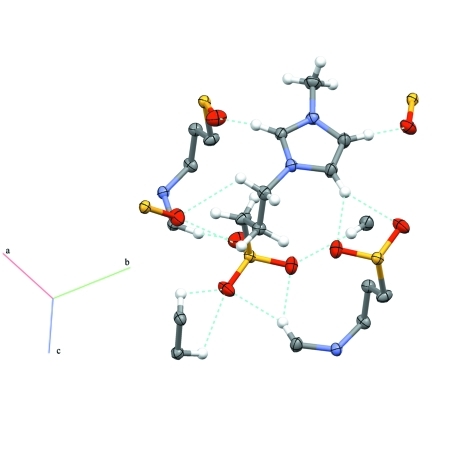
Close contacts in compound I.

**Fig. 3.**
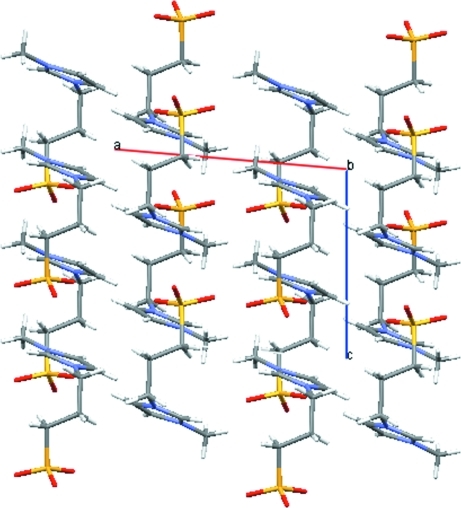
Packing diagram along the b axis.

**Fig. 4.**
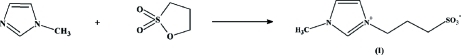
Reaction scheme.

## supplementary crystallographic information

### Comment

Ionic liquids (ILs) have proven to be highly versatile materials with an ever expanding suite of chemical applications. An application that has recently shown great promise is the use of functionalized ILs as Brønsted acid catalysts for organic reactions (Cole *et al.*, 2002). These IL catalysts are most commonly prepared by the reaction of 1-methylimidazolium-3-alkyl sulfonate zwitterion with an acid that has a pKa low enough to protonate the sulfonate group (Yoshizawa *et al.*, 2001; Cole *et al.*, 2002). The activity of the IL (e.g. the effectiveness of proton transfer) is significantly impacted by the structure and interactions of the zwitterion. It has been shown that the local structure of ILs is often conserved on transition from the solid state to the liquid state (Triolo *et al.*, 2006; Henderson *et al.*, 2007; Reichert *et al.*, 2007). Thus, a structural analysis of the zwitterion, 1-methylimidaolium-3-propanesulfonate (I) might provide valuable insight into the activity of the Brønsted acid IL catalyst.

The asymmetric unit of the title compound is presented in Figure 1. The dominant intermolecular interactions are Coulombic in nature and are through the charged centers of the zwitterion: the imidazolium ring and the sulfonate group (Fig. 2). The negative charged sulfonate group is surrounded by four imidazolium head groups forming six close contacts (Table 1). The interactions of the imidazolium ring hydrogen atoms with the sulfonate group establish two three-dimensional R22(16) rings. The packing along the b axis (Fig. 3) shows the zwitterions arranged in columns along the c axis. The head-to-tail orientation maximizes the polar interaction and minimizes cation-cation and anion-anion repulsions.

### Experimental

Compound I was synthesized following the procedure for similar zwitterionic compounds published by (Yoshizawa *et al.*, 2001). 1,3-Propane sultone (25 g, 0.122 mol) was added dropwise to a solution of 1-methylimidazole (10 g, 0.122 mol) in acetone (40 ml) and stirred, then cooled on an ice bath overnight. A white precipitate was recovered from the reaction solution through filtration and washing with acetone. The product was then dried under vacuum giving a white solid (m.p. 482 K). A colourless crystal suitable for single crystal X-ray diffraction was retrieved from the dried product.

### Refinement

(type here to add refinement details)

### Crystal data

**Table:**

C_7_H_12_N_2_O_3_S	*F*(000) = 432
*M**_r_* = 204.25	*D*_x_ = 1.481 Mg m^−^^3^
Monoclinic, *P*2_1_/*c*	Melting point: 482 K
Hall symbol: -P 2ybc	Mo *K*α radiation, λ = 0.71073 Å
*a* = 9.8164 (4) Å	Cell parameters from 4851 reflections
*b* = 11.7421 (5) Å	θ = 3.1–41.1°
*c* = 7.9769 (3) Å	µ = 0.33 mm^−^^1^
β = 94.878 (2)°	*T* = 296 K
*V* = 916.13 (6) Å^3^	Plate, colourless
*Z* = 4	0.29 × 0.28 × 0.13 mm

### Data collection

**Table:**

Bruker SMART CCD area-detector diffractometer	8299 independent reflections
Radiation source: fine-focus sealed tube	5726 reflections with *I* > 2σ(*I*)
graphite	*R*_int_ = 0.033
phi and ω scans	θ_max_ = 47.1°, θ_min_ = 3.1°
Absorption correction: for a sphere (*SADABS*; Bruker, 2007)	*h* = −20→13
*T*_min_ = 0.910, *T*_max_ = 0.958	*k* = −19→24
21327 measured reflections	*l* = −16→16

### Refinement

**Table:**

Refinement on *F*^2^	Primary atom site location: structure-invariant direct methods
Least-squares matrix: full	Secondary atom site location: difference Fourier map
*R*[*F*^2^ > 2σ(*F*^2^)] = 0.041	Hydrogen site location: inferred from neighbouring sites
*wR*(*F*^2^) = 0.120	All H-atom parameters refined
*S* = 1.03	*w* = 1/[σ^2^(*F*_o_^2^) + (0.0574*P*)^2^ + 0.0483*P*] where *P* = (*F*_o_^2^ + 2*F*_c_^2^)/3
8299 reflections	(Δ/σ)_max_ = 0.002
166 parameters	Δρ_max_ = 0.55 e Å^−^^3^
0 restraints	Δρ_min_ = −0.47 e Å^−^^3^

### Special details

**Table:**

Geometry. All esds (except the esd in the dihedral angle between two l.s. planes) are estimated using the full covariance matrix. The cell esds are taken into account individually in the estimation of esds in distances, angles and torsion angles; correlations between esds in cell parameters are only used when they are defined by crystal symmetry. An approximate (isotropic) treatment of cell esds is used for estimating esds involving l.s. planes.
Refinement. Refinement of *F*^2^ against ALL reflections. The weighted *R*-factor wR and goodness of fit *S* are based on *F*^2^, conventional *R*-factors *R* are based on *F*, with *F* set to zero for negative *F*^2^. The threshold expression of *F*^2^ > σ(*F*^2^) is used only for calculating *R*-factors(gt) etc. and is not relevant to the choice of reflections for refinement. *R*-factors based on *F*^2^ are statistically about twice as large as those based on *F*, and *R*- factors based on ALL data will be even larger.

### Fractional atomic coordinates and isotropic or equivalent isotropic displacement parameters (Å^2^)

**Table:**

	*x*	*y*	*z*	*U*_iso_*/*U*_eq_	
S1	0.288447 (16)	0.459431 (14)	0.711035 (19)	0.01929 (4)	
O1	0.18103 (7)	0.54452 (5)	0.71911 (8)	0.02993 (12)	
O2	0.42331 (6)	0.50523 (8)	0.76230 (9)	0.03616 (15)	
O3	0.25705 (8)	0.35528 (5)	0.79823 (8)	0.03407 (14)	
N3	0.17238 (6)	0.51000 (5)	0.16133 (7)	0.01989 (9)	
N1	0.28516 (6)	0.65793 (5)	0.08981 (8)	0.02208 (9)	
C2	0.29151 (7)	0.54584 (6)	0.11322 (8)	0.02096 (10)	
H2A	0.3704 (14)	0.5037 (13)	0.1080 (19)	0.037 (3)*	
C4	0.08588 (8)	0.60254 (6)	0.16753 (10)	0.02580 (12)	
H4A	−0.0036 (14)	0.5991 (11)	0.2077 (16)	0.034 (3)*	
C5	0.15675 (8)	0.69513 (7)	0.12222 (11)	0.02724 (13)	
H5A	0.1323 (14)	0.7688 (12)	0.1137 (17)	0.037 (3)*	
C6	0.39573 (9)	0.72763 (8)	0.03336 (12)	0.03149 (15)	
H6A	0.3932 (15)	0.8024 (14)	0.0813 (19)	0.044 (4)*	
H6B	0.4809 (19)	0.6923 (15)	0.063 (2)	0.061 (5)*	
H6C	0.3820 (17)	0.7417 (17)	−0.081 (2)	0.067 (5)*	
C7	0.14604 (8)	0.39419 (6)	0.21968 (8)	0.02303 (11)	
H7A	0.0552 (13)	0.3717 (11)	0.1696 (15)	0.027 (3)*	
H7B	0.2156 (12)	0.3490 (11)	0.1741 (15)	0.026 (3)*	
C8	0.15267 (7)	0.38976 (6)	0.41105 (8)	0.02112 (10)	
H8A	0.0853 (14)	0.4389 (12)	0.4510 (18)	0.033 (3)*	
H8B	0.1301 (13)	0.3105 (12)	0.4405 (17)	0.036 (3)*	
C9	0.29159 (7)	0.42456 (8)	0.49418 (9)	0.02574 (12)	
H9A	0.3243 (16)	0.4859 (13)	0.444 (2)	0.043 (4)*	
H9B	0.3600 (16)	0.3658 (13)	0.4885 (18)	0.043 (4)*	

### Atomic displacement parameters (Å^2^)

**Table:**

	*U*^11^	*U*^22^	*U*^33^	*U*^12^	*U*^13^	*U*^23^
S1	0.01791 (6)	0.02075 (7)	0.01894 (6)	−0.00086 (5)	0.00002 (4)	0.00099 (4)
O1	0.0309 (3)	0.0258 (2)	0.0325 (3)	0.0079 (2)	−0.0007 (2)	−0.00600 (19)
O2	0.0218 (2)	0.0569 (4)	0.0287 (3)	−0.0109 (3)	−0.0044 (2)	−0.0016 (3)
O3	0.0510 (4)	0.0231 (2)	0.0281 (2)	−0.0029 (2)	0.0032 (2)	0.00664 (19)
N3	0.0201 (2)	0.01929 (19)	0.02006 (19)	0.00022 (16)	0.00055 (16)	−0.00017 (15)
N1	0.0208 (2)	0.0222 (2)	0.0233 (2)	−0.00089 (18)	0.00185 (17)	0.00077 (17)
C2	0.0201 (2)	0.0225 (2)	0.0204 (2)	0.00164 (19)	0.00209 (18)	0.00037 (18)
C4	0.0200 (3)	0.0237 (3)	0.0339 (3)	0.0023 (2)	0.0034 (2)	0.0011 (2)
C5	0.0245 (3)	0.0214 (3)	0.0361 (3)	0.0030 (2)	0.0042 (2)	0.0014 (2)
C6	0.0295 (4)	0.0310 (3)	0.0347 (4)	−0.0069 (3)	0.0068 (3)	0.0048 (3)
C7	0.0289 (3)	0.0190 (2)	0.0207 (2)	−0.0029 (2)	−0.0008 (2)	−0.00147 (18)
C8	0.0208 (2)	0.0220 (2)	0.0204 (2)	−0.00162 (19)	0.00092 (18)	−0.00033 (18)
C9	0.0180 (2)	0.0392 (4)	0.0200 (2)	0.0002 (2)	0.00188 (18)	−0.0015 (2)

### Geometric parameters (Å, °)

**Table:**

S1—O3	1.4529 (6)	C5—H5A	0.899 (14)
S1—O2	1.4550 (6)	C6—H6A	0.959 (16)
S1—O1	1.4580 (6)	C6—H6B	0.945 (19)
S1—C9	1.7805 (7)	C6—H6C	0.924 (19)
N3—C2	1.3298 (9)	C7—C8	1.5233 (9)
N3—C4	1.3825 (9)	C7—H7A	0.982 (13)
N3—C7	1.4673 (9)	C7—H7B	0.961 (12)
N1—C2	1.3301 (9)	C8—C9	1.5207 (10)
N1—C5	1.3791 (10)	C8—H8A	0.952 (14)
N1—C6	1.4607 (10)	C8—H8B	0.990 (14)
C2—H2A	0.923 (14)	C9—H9A	0.898 (16)
C4—C5	1.3562 (11)	C9—H9B	0.967 (16)
C4—H4A	0.961 (14)		
			
O3—S1—O2	113.68 (5)	H6A—C6—H6B	110.8 (14)
O3—S1—O1	111.84 (4)	N1—C6—H6C	110.7 (11)
O2—S1—O1	112.24 (5)	H6A—C6—H6C	102.9 (15)
O3—S1—C9	107.06 (4)	H6B—C6—H6C	112.0 (15)
O2—S1—C9	105.52 (4)	N3—C7—C8	110.85 (5)
O1—S1—C9	105.84 (4)	N3—C7—H7A	107.3 (7)
C2—N3—C4	108.62 (6)	C8—C7—H7A	111.0 (7)
C2—N3—C7	124.60 (6)	N3—C7—H7B	104.0 (7)
C4—N3—C7	126.28 (6)	C8—C7—H7B	112.9 (7)
C2—N1—C5	108.65 (6)	H7A—C7—H7B	110.4 (10)
C2—N1—C6	124.78 (7)	C9—C8—C7	112.84 (6)
C5—N1—C6	126.54 (7)	C9—C8—H8A	108.3 (8)
N3—C2—N1	108.77 (6)	C7—C8—H8A	110.0 (8)
N3—C2—H2A	127.3 (9)	C9—C8—H8B	111.1 (8)
N1—C2—H2A	123.6 (9)	C7—C8—H8B	106.2 (8)
C5—C4—N3	106.86 (7)	H8A—C8—H8B	108.4 (11)
C5—C4—H4A	128.8 (8)	C8—C9—S1	113.36 (5)
N3—C4—H4A	124.1 (8)	C8—C9—H9A	111.2 (10)
C4—C5—N1	107.09 (7)	S1—C9—H9A	106.8 (10)
C4—C5—H5A	130.7 (9)	C8—C9—H9B	112.9 (9)
N1—C5—H5A	122.2 (9)	S1—C9—H9B	106.2 (9)
N1—C6—H6A	110.2 (9)	H9A—C9—H9B	105.9 (13)
N1—C6—H6B	110.1 (11)		
			
C2—N3—C7—C8	100.05 (8)	C4—N3—C7—C8	−70.97 (8)
N3—C7—C8—C9	−60.48 (8)	N3—C7—C8—C9	−60.48 (8)
C7—C8—C9—S1	163.00 (5)		

### Hydrogen-bond geometry (Å, °)

**Table:**

*D*—H···*A*	*D*—H	H···*A*	*D*···*A*	*D*—H···*A*
C2—H2A···O2^i^	0.923 (14)	2.197 (14)	2.9512 (9)	138.4 (12)
C5—H5A···O1^ii^	0.899 (14)	2.381 (15)	3.1573 (10)	144.6 (12)
C4—H4A···O1^iii^	0.961 (14)	2.528 (14)	3.3268 (11)	140.5 (11)
C4—H4A···O3^iii^	0.961 (14)	2.541 (14)	3.4364 (11)	155.0 (11)
C7—H7B···O3^iv^	0.961 (12)	2.613 (12)	3.1693 (10)	117.2 (9)
C8—H8B···O3^iv^	0.990 (14)	2.621 (13)	3.2086 (9)	118.1 (9)

Symmetry codes: (i) −*x*+1, −*y*+1, −*z*+1; (ii) *x*, −*y*+3/2, *z*−1/2; (iii) −*x*, −*y*+1, −*z*+1; (iv) *x*, −*y*+1/2, *z*−1/2.
